# Echocardiography in the Era of Multimodality Cardiovascular Imaging

**DOI:** 10.1155/2013/310483

**Published:** 2013-06-26

**Authors:** Benoy Nalin Shah

**Affiliations:** ^1^Cardiovascular Biomedical Research Unit, Royal Brompton Hospital, Sydney Street, London SW3 6NP, UK; ^2^National Heart & Lung Institute, Imperial College, London SW7 2AZ, UK

## Abstract

Echocardiography remains the most frequently performed cardiac imaging investigation and is an invaluable tool for detailed and accurate evaluation of cardiac structure and function. Echocardiography, nuclear cardiology, cardiac magnetic resonance imaging, and cardiovascular-computed tomography comprise the subspeciality of cardiovascular imaging, and these techniques are often used together for a multimodality, comprehensive assessment of a number of cardiac diseases. This paper provides the general cardiologist and physician with an overview of state-of-the-art modern echocardiography, summarising established indications as well as highlighting advances in stress echocardiography, three-dimensional echocardiography, deformation imaging, and contrast echocardiography. Strengths and limitations of echocardiography are discussed as well as the growing role of real-time three-dimensional echocardiography in the guidance of structural heart interventions in the cardiac catheter laboratory.

## 1. Introduction

The beginning of the twenty-first century in cardiovascular imaging has been dominated by one theme: “multimodality.” It is currently the buzz word of imaging, as the “traditional” techniques of echocardiography and nuclear cardiology have been complimented by the more “modern” techniques of cardiovascular magnetic resonance (CMR) and cardiac-computed tomography (CCT). Cardiovascular imaging has seen an unprecedented growth in demand, fuelled by technological advances and wider availability of these modalities [1]. Each of these techniques has strengths and weaknesses (Table 1) and, in combination, they are used widely in the management of patients with coronary heart disease, valvular heart diseases, heart failure, congenital heart disease, and arrhythmias. The aim of this review is to provide a comprehensive overview of contemporary echocardiography for the general cardiologist and physician, summarising established indications with the supporting evidence and also highlighting recent technological advances within the subspeciality, particularly in stress three-dimensional echocardiography, deformation imaging and contrast echocardiography, and the emerging role of real-time echocardiography for guidance of interventional procedures.

## 2. The Evolution of Cardiac Ultrasound

Echocardiography has witnessed dramatic improvements in technology over the past 25 years. Aside from the plain chest X-ray, it remains the primary—and thus most frequently performed—cardiac imaging investigation. A wealth of structural (anatomical) and haemodynamic (physiological) information can be ascertained from one examination. From the one-dimensional M-mode echocardiogram to real-time three-dimensional imaging, an echocardiographic study in 2013 can reveal physiology and pathology in detail previously unimaginable.

The numerous modalities within echocardiography furnish the scanning sonographer or physician with a wide array of technologies for complete assessment of cardiac morphology and function. The high temporal resolution of M-mode echocardiography permits accurate depiction of rapidly moving structures and retains relevance in today's practice. 2D transthoracic echocardiography (TTE) forms the basis of visual assessment of cardiac chambers and valves and permits identification of structural abnormalities. Doppler echocardiography, comprising spectral Doppler (pulsed wave and continuous wave) and colour Doppler imaging, identifies the direction, velocity, amplitude, and timing of blood flow through the heart. This information has an enormous number of uses—Doppler echocardiography remains the cornerstone of evaluating the severity of stenotic and regurgitant valve diseases, estimating LV filling pressures (and thus confirming the diagnosis of diastolic dysfunction and, in symptomatic patients, heart failure with preserved ejection fraction), detection of intracavity and subvalvular obstruction in hypertrophic cardiomyopathy, and for identification of intracardiac shunts (e.g., septal defects). 

**Figure 1:**
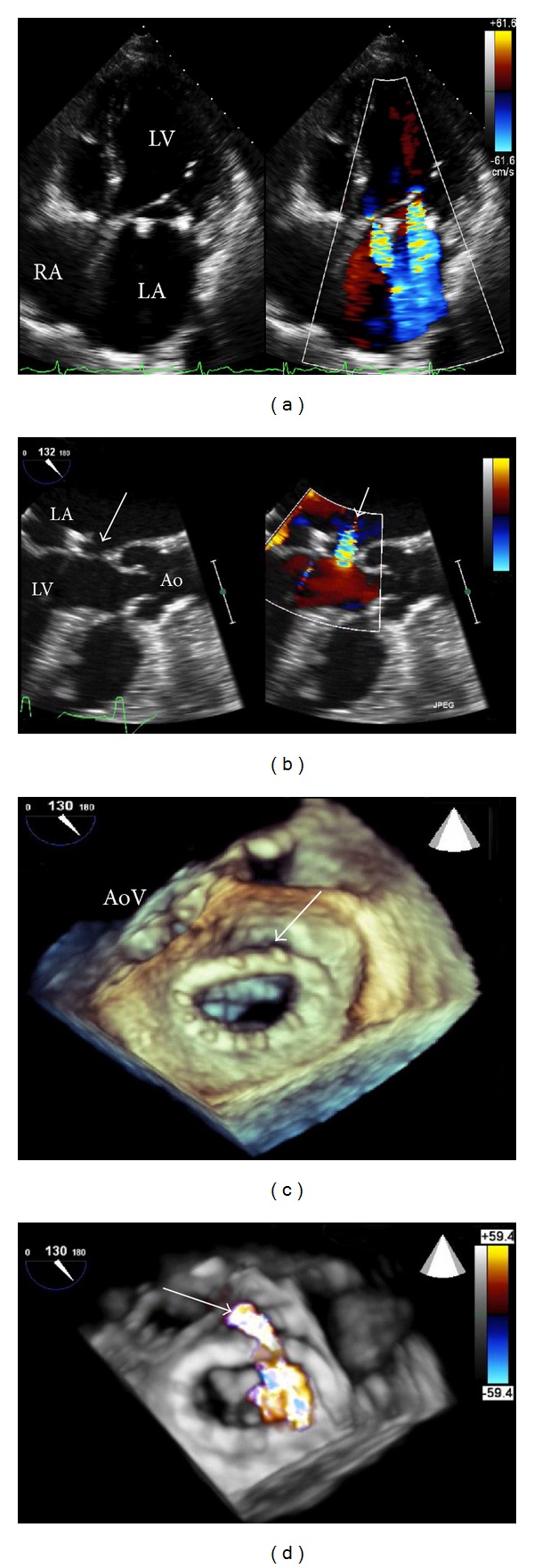
A patient with previous mitral valve repair attended with increasing breathlessness. 2D-TTE revealed two jets of mitral regurgitation (panel (a)). 2D-TOE suggested the presence of a paravalvular leak (panel (b), see arrows). Three-dimensional TOE revealed dehiscence of the posterior aspect of the mitral annuloplasty ring (panel (c), see arrow), and real-time 3D colour during TOE confirmed the presence of a reguritant jet (arrow) through this defect (panel (d)). The patient subsequently underwent redo mitral valve surgery. (LA: left atrium; RA: right atrium; LV: left ventricle; Ao: aorta; AoV: aortic valve).

Transoesophageal echocardiography (TOE) permits detailed views of most cardiac structures and has a very wide number of indications, from exclusion of atrial appendage thrombus prior to cardioversion through to investigation of suspected cardioembolic stroke [2]. In most centres, TOE remains the imaging technique of choice for precise assessment of mechanisms and severity of valvular heart disease and their associated complications (Figure 1). Although semi-invasive in nature, TOE is generally well tolerated and has an excellent safety profile [3, 4]. 

## 3. Specific Advances

### 3.1. Stress Echocardiography

Stress echocardiography (SE) has progressed significantly over the past three decades to become one of the most widely utilised imaging techniques for evaluation of patients with known or suspected coronary artery disease (CAD). Once heavily criticised for being too “operator dependent,” several technical advances have dramatically improved the accuracy—and reduced variability—of SE. These include firstly the advent of tissue harmonic imaging, which was shown to improve diagnostic accuracy for detection of CAD over fundamental frequency imaging in exercise [5] and dobutamine [6] SE, secondly the introduction of digital cine loop acquisition with side-by-side display of rest and stress images for ease of comparison, and thirdly, and possibly most importantly, the availability of ultrasound contrast agents for improved visualisation of the endocardial border and LV cavity during rest and stress imaging [7].

SE has numerous advantages as a stress technique but also some disadvantages. Its advantages include the ubiquitous availability of echocardiography machines and thus the low infrastructure cost, lack of exposure to ionising radiation, safety, multiple stressor options (physiological (treadmill or bicycle) or pharmacological (inotropes or vasodilators)), portability, and the substantial supporting evidence base accrued over the past 20 years. The option of performing physiological stress is important, as this should always be the preferred stress technique in an attempt to reproduce patient's symptoms and also determine their functional aerobic capacity. Disadvantages include a lower sensitivity than perfusion techniques and suboptimal image quality in some patients despite use of ultrasound contrast.

In the United Kingdom, SE is now—along with myocardial perfusion scintigraphy and CMR—recommended over exercise electrocardiography (ex-ECG) for the investigation of patients with chest pain suspected to be angina pectoris [8]. A randomised controlled trial showed that SE is more cost-effective than ex-ECG for establishing the diagnosis of CAD in patients hospitalised with chest pain [9], and a recent large, real-world study of the incorporation of SE into a chest pain unit has confirmed the feasibility and prognostic value of this approach in routine practice [10]. 

There is also now growing appreciation of the potential clinical value of SE for “noncoronary” applications (see Table 2), in particular the dynamic assessment of valvular heart diseases. These patients have traditionally been evaluated by resting echocardiography only, although their symptoms are typically induced by exertion. Exercise echocardiography can be used to risk stratify asymptomatic patients with severe aortic stenosis (AS). Stress-induced increases in mean transaortic gradient [11, 12] and pulmonary pressures [13] are associated with worse outcome. Furthermore, low-dose dobutamine stress echocardiography is frequently used to differentiate true severe AS from “pseudosevere” AS in patients with low-flow low-gradient aortic stenosis [14]. 

**Figure 2:**
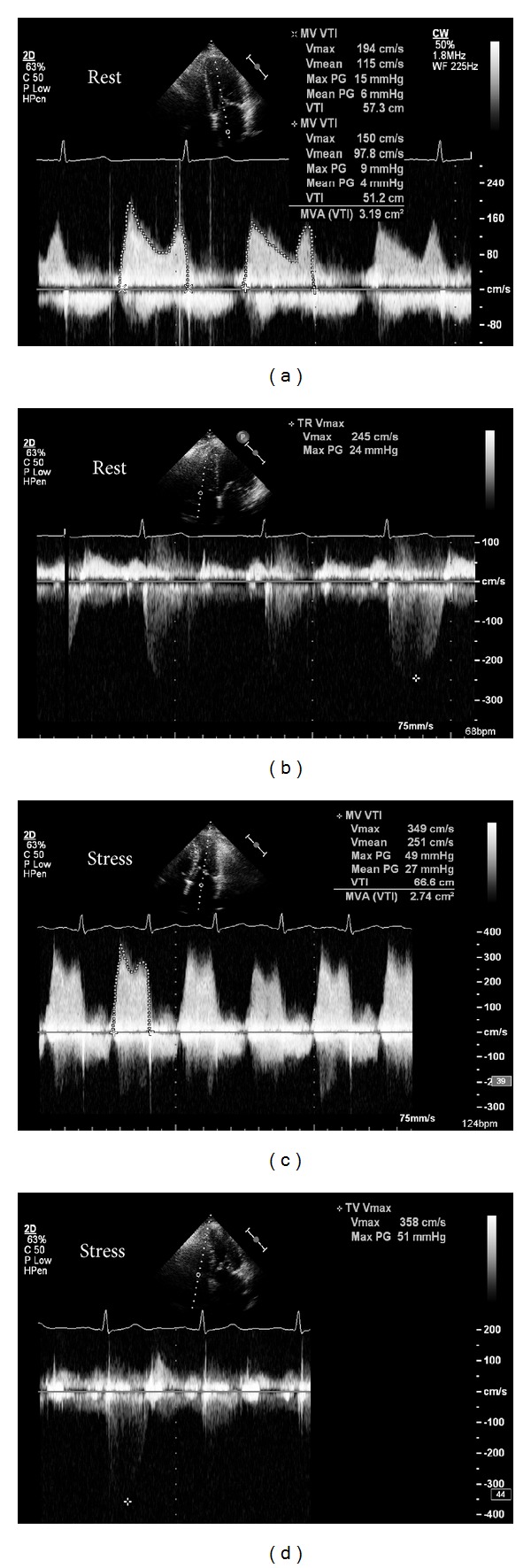
A patient with moderate mitral stenosis but worsening exertional dyspnoea underwent exercise SE. At rest, mean transmitral gradient was 5 mm Hg (a), and transtricuspid pressure gradient was 24 mm Hg (b). After 7-minute BRUCE protocol treadmill exercise, mean transmitral gradient significantly increased to 27 mm Hg (c) and transtricuspid pressure gradient had more than doubled to 51 mm Hg (d). The patient subsequently underwent balloon mitral valvuloplasty with excellent symptomatic relief.

Exercise SE is also helpful in certain patients with mitral valve disease. Patients with mitral stenosis (MS) who have symptoms disproportionate to the degree of MS at rest (e.g., marked exertional dyspnoea but only moderate MS at rest) should undergo exercise SE—an increase in mean transmitral gradient to >15 mm Hg or pulmonary artery pressure to >60 mm Hg is an indication for valve intervention (Figure 2). The dynamic nature of ischaemic mitral regurgitation (MR) was previously shown in a quantitative Doppler echocardiography study, using exercise stress in patients with ischaemic cardiomyopathy (ICM) recently admitted with pulmonary oedema and comparing with ICM patients without pulmonary oedema [15]. Patients with recent pulmonary oedema had significantly greater increases in MR and pulmonary pressures during exercise than those without pulmonary oedema. The same group has also demonstrated the clinical value of exercise SE in patients with degenerative MR [16]. A recent analysis clearly indicated the value of SE in valve disease in daily clinical practice and also suggested expansion of SE in valve disease beyond that indicated by current guidelines [17].

**Figure 3:**
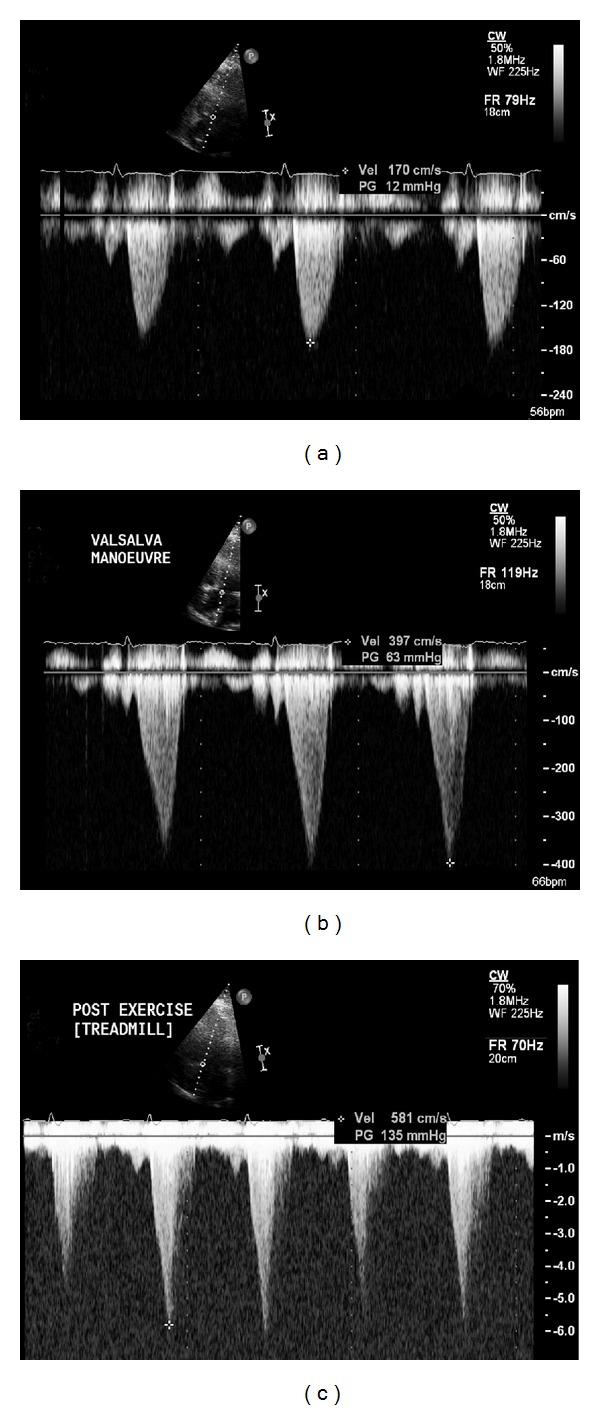
A 47-year-old man with hypertrophic cardiomyopathy underwent exercise SE. At rest, peak gradient across the aortic valve was 12 mm Hg (panel (a)). Repeat assessment during a held Valsalva manoeuvre revealed that the peak gradient was now 63 mm Hg (panel (b)), which indicates that exercise-induced LVOT obstruction is highly likely. This was indeed confirmed that at peak stress, the maximum gradient had increased to 135 mm Hg (panel (c)). Note the late systolic peaking of the Doppler profile in panels (b) and (c), indicative of dynamic rather than fixed outflow tract obstruction. The patient was commenced on beta-blocker therapy following the test results.

Aside from valvular heart disease, exercise SE can also be used in patients with hypertrophic cardiomyopathy to reveal dynamic left ventricular outflow tract obstruction (Figure 3) [18]. Finally, myocardial contractile reserve, determined by SE, can identify those patients that will experience LV reverse remodelling following cardiac resynchronisation therapy [19].

### 3.2. Three-Dimensional Echocardiography

Three-dimensional (3D) echocardiography is widely perceived as a novel technique, but the initial work on 3D echocardiography dates back three decades [20, 21]. However, significant technical advances have reduced the size of the transducer, improved speed of image processing, and, importantly, allowed real-time 3D imaging. This has been made possible with the advent of fully sampled matrix array transducer technology [22].

3D-TTE has numerous proven uses in clinical practice. It has been shown to be superior to 2D-TTE and equal to CMR for assessment of ventricular volumes [23], cardiac mass [24], and calculation of ejection fraction [25]. The latter is especially useful clinically in patients that require serial EF assessment (e.g., cancer patients receiving chemotherapeutic agents with potential cardiotoxicity) as 2D-TTE can suffer from underestimation of true volumes due to chamber foreshortening. 3D-TTE has also been used to assess ventricular mechanical dyssynchrony [26], and, of clinical relevance, dyssynchrony demonstrated by real-time 3D-TTE can predict long-term response to cardiac resynchronization therapy [27]. 3D-TTE has also been proved to be valuable in assessment of valvular heart disease—it can provide information previously only easily determined by TOE (e.g., mitral valve scallop assessment in mitral regurgitation). The accuracy of calculation of aortic valve area in aortic stenosis has also been shown to be improved by using 3D-TTE due to more precise assessment of outflow tract dimensions [28].

The most significant advance, however, has been in 3D-TOE [29]. Real-time, 3D zoom, and live 3D imaging provide unique views of the heart and allow superior detection of pathology, especially valvular diseases. 3D echocardiography is most valuable in cases of complex pathology. A simple P2 prolapse of the mitral valve is usually evident from 2D imaging, but a combination of flail scallop(s) and prolapse +/− chordal rupture may be more challenging on 2D, and any confusion is usually dispelled with 3D imaging. As discussed in the following, the feasibility of real-time 3D-TOE has made it an essential adjunct in guiding invasive procedures in the catheter laboratory also.

### 3.3. Deformation Imaging: “Myocardial Mechanics”

The complex orientation of myocardial fibres—radial, circumferential, and longitudinal—has been recognised for a long time. The theory that tissue motion, as opposed to endocardial excursion, is important in assessment ventricular function is also not new—M-mode echocardiography was used to relate myocardial velocity to ventricular function in the 1970s [30].

Tissue Doppler imaging (TDI) and speckle tracking echocardiography (STE) are techniques that permit detailed analysis of regional as well as global cardiac function through unique temporal and spatial data processing. TDI deliberately filters out the low amplitude, high frequency signals from the blood-pool in order to allow analysis of the high amplitude, and low frequency signals from myocardium itself [31]. TDI is most frequently used to assist in determining diastolic function and LV filling pressures by means of the *E*/*E*′ ratio [32] (where *E*′ is the early motion of the mitral annulus back towards the cardiac base from the apex). The longitudinal function of both ventricles is also assessed with TDI, where *S*′ represents the peak systolic velocity of the left or right ventricle. These parameters have become routine measurements in clinical practice due to a combination of their reproducibility and diagnostic and prognostic importance.

**Figure 4:**
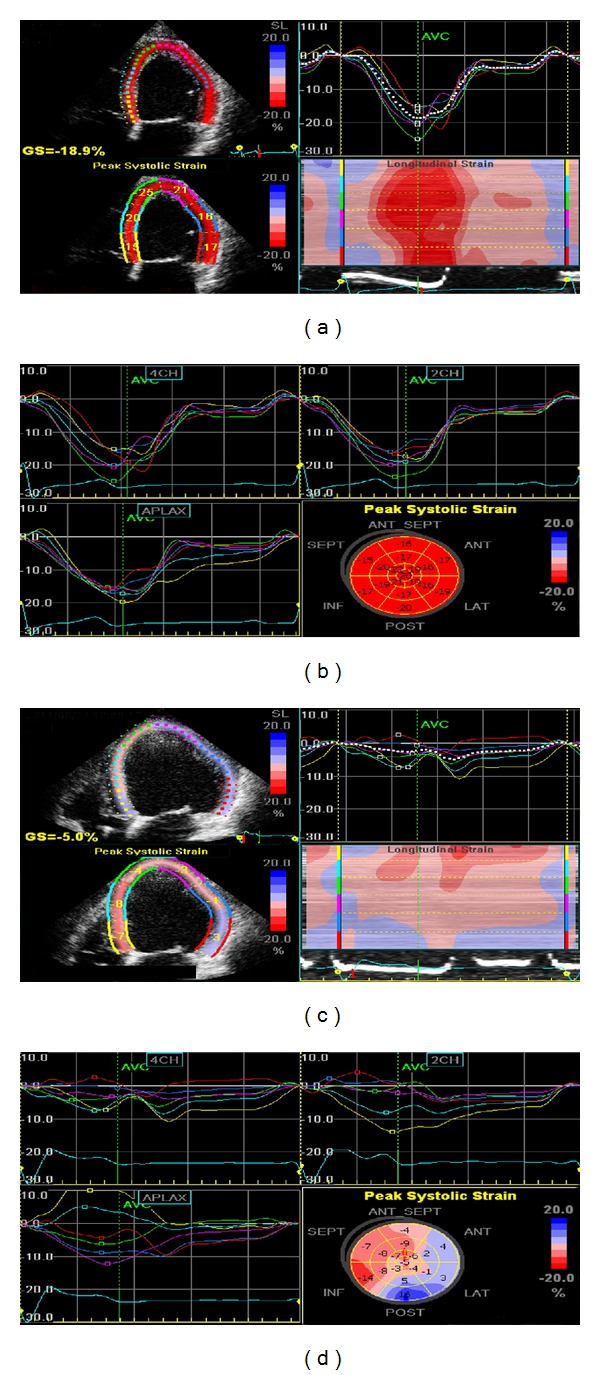
Examples of strain imaging using speckle tracking echocardiography. On the top row are images from a normal healthy volunteer (apical 4-chamber view on the left and summary strain scores with bulls-eye plot on the right), and on the bottom row are corresponding images from a patient with severe ischaemic cardiomyopathy. Note that the healthy volunteer has normal longitudinal strain in all segments with a global strain score of −18.9%, whereas the cardiomyopathy patient has highly abnormal strains with a significantly depressed global strain score of just −5.0%.

STE is based on the principle of following (or tracking) the unique “speckle pattern” of a myocardial segment through the cardiac cycle (see Figure 4). One advantage of STE is that it removes the possibility of falsely designating myocardium as having normal function when “velocity” is in fact due to tethering (e.g., adjacent scar) or translational motion due to movement of the whole heart (both potential limitations of TDI [33]). Both TDI and STE can be used to measure myocardial strain, a dimensionless quantity defined as the unit change in length of an object relative to its original length. Strain rate is its derivative with respect to time (i.e., rate of change of strain and usually expressed as 1/sec (or sec^−1^)). In normal myocardial segments, there is shortening in systole (negative strain and strain rates) and lengthening in diastole (positive strain and strain rates) as cardiomyocytes return to their resting state [33].

A wealth of data has been accumulated on the potential clinical value of strain and strain rate assessment in a number of cardiac diseases [34]. Normal ranges have also been defined [35]. STE-derived strain imaging has been shown to have additive benefit for detection of CAD in patients undergoing dobutamine SE [36], and the superior ability of strain imaging to predict outcome in patients undergoing dobutamine SE has also been shown [37]. Strain imaging is an accurate technique for detecting viable myocardium, particularly when strain parameters are assessed at rest and following low-dose dobutamine SE [38, 39]. Strain imaging has also been studied extensively for detecting dyssynchrony and thus predicting response to cardiac resynchronisation therapy [40]. Recently, it was shown that targeted placement of the LV pacing lead to the site of latest mechanical activation—as elucidated by STE—was associated with favourable reverse remodelling, fewer repeat hospitalizations, and improved functional capacity compared to patients with standard placement of LV leads [41]. TDI can also be used to assess cardiac dyssynchrony. This has become a controversial topic; however, as the PROSPECT trial [42] suggested no echocardiographic parameter(s) could accurately predict favourable response to biventricular pacing, though numerous authorities worldwide were quick to highlight surprisingly fundamental flaws in the trial's methodology [43]. 

Potentially of great clinical relevance is the fact that STE can reveal subclinical LV dysfunction in hearts with apparently normal systolic function, as measured by EF, and thus could have a role in detecting cardiac involvement in systemic diseases [44], unusual or rare conditions [45], and also for detecting early evidence of cardiotoxicity in cancer patients receiving chemotherapy [46]. At present, strain and strain rate imaging remains predominantly research tools but shows considerable promise for translation into clinical practice. The limitations of STE include the requirement of good image quality and variability between different software and hardware manufacturers.

### 3.4. Contrast Echocardiography

Ultrasound contrast agents (UCAs) consist of gas-filled microspheres which display unique acoustic properties when exposed to ultrasound waves. Contrast bubbles resonate when exposed to an ultrasound wave. This is predominantly a nonlinear oscillation (i.e., expansion and contraction of the bubble are not equal) at the ultrasound frequencies used in diagnostic imaging. Microbubbles are several million times more effective at scattering sound than red blood cells, resulting in a greatly enhanced “blood pool” signal [47]. The blood pool agents developed consist of a gaseous material encapsulated within a stabilizing outer shell. These microbubbles are typically slightly smaller than erythrocytes, allowing free passage within the circulation and effectively acting as red cell tracers [48].

**Figure 5:**
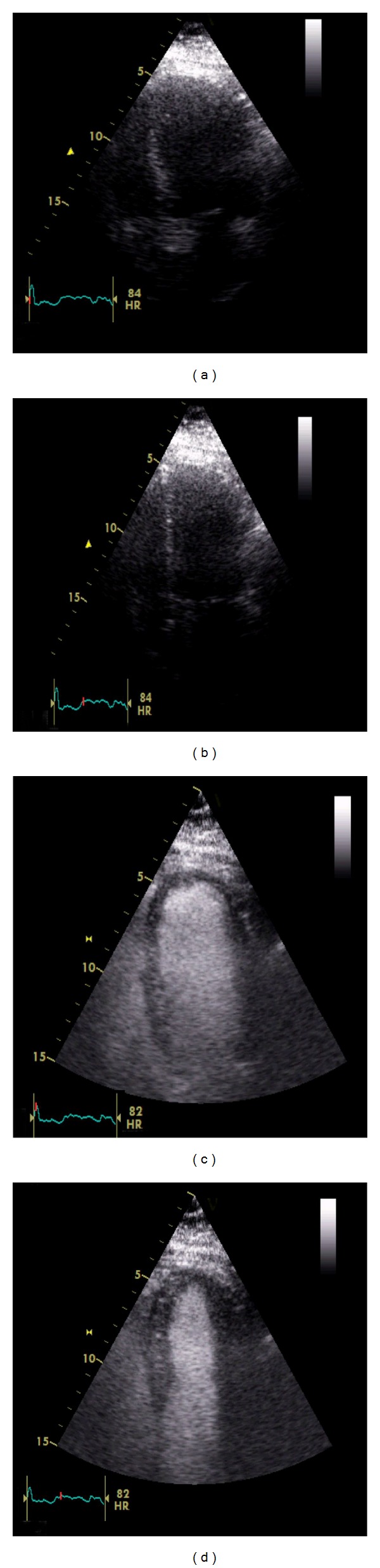
Apical 4-chamber images taken before ((a) and (b)) and after ((c) and (d)) ultrasound contrast injection in a patient attending for routine transthoracic echocardiography. At rest, endocardial definition at end diastole (a) and end systole (b) was poor and did not permit quantitation of ejection fraction or assessment of regional (segmental) function. Following a single bolus injection of contrast, endocardial border visualisation was significantly improved at end diastole (c) and end systole (d), allowing regional and global systolic assessments.

The first generation UCAs consisted of air surrounded by an albumin shell [49]. Unfortunately, these agents had a very short lifespan in vivo and thus had limited utility in diagnostic tests. The second generation of UCAs addressed this issue by changing both components of microbubbles—the air was replaced by inert gases with high molecular weight, high density, and low solubility, such as sulphur hexafluoride (SonoVue) or perfluorocarbons (e.g., Optison or Definity). The outer shell is now made using phospholipids, which also confer greater stability. They are used to produce a stronger ultrasonic signal than that generated by tissues and, thus, are most commonly used to improve image quality (Figure 5). 

There is ample evidence demonstrating that UCAs improve estimation of global [50] and regional [51] LV systolic functions. UCAs improve the accuracy of measurement of LV volumes and ejection fraction also [52, 53]. As UCAs improve overall image quality, they also help detect structural abnormalities such as noncompaction cardiomyopathy [54], apical hypertrophic cardiomyopathy [55], and left ventricular thrombus [56]. Their biggest use, however, is in stress echocardiography. The use of contrast significantly reduces the number of “uninterpretable” studies [7] and thus is cost-effective as it reduces additional testing [57]. This has helped establish stress echocardiography as a front-line investigation in assessment of patients with known or suspected coronary artery disease.

The majority (90%) of the blood within the myocardial walls reside within capillaries [58]. Thus the intensity of the contrast signal, when the myocardium is fully saturated with contrast, reflects the concentration of microbubbles within myocardial capillaries [59] and, consequently, capillary or myocardial blood volume. Appreciation of this fact led to the recognition that echocardiography could be used to assess myocardial perfusion, a technique now known as myocardial contrast echocardiography (MCE). A high intensity (high mechanical index) “flash” or impulse is used to destroy the microbubbles in the myocardial capillaries, and their rate of replenishment is observed. Delayed replenishment manifests as a perfusion defect and is the hallmark of ischaemia during MCE. The first papers on MCE dates back to three decades [60, 61], and since then, it has accumulated a large evidence base to support its use in the detection of CAD [62]. Finally, as presence of contrast denotes vascularity, UCAs can also be used in patients with intracardiac masses in order to distinguish between avascular masses (e.g., thrombus) and vascular masses such as tumours [63]. 

## 4. “Interventional Echocardiology”: Dawn of a New Super-Specialty?

The advances in 3D echocardiography—in particular the advent of a fully sampled matrix array transducer allowing real-time 3D-TOE—has brought the echocardiologist to the forefront of catheter-lab-based procedures in structural heart disease. Real-time 3D-TOE is now considered by many a pre-requisite for guidance of percutaneous treatment of aortic stenosis (transcatheter aortic valve implantation (TAVI)), mitral regurgitation (e.g., MitraClip devices), closure of the left atrial appendage and of atrial septal defects (ASDs and PFOs). The incremental value of 3D-TOE over 2D-TOE during TAVI procedures was recently demonstrated in a large single-centre study [64]. Indeed, real-time 3D-TOE is expanding its use further and, for example, has been used to guide electrophysiological procedures such as ablation of atrial flutter and fibrillation (helping reduce significantly the use of fluoroscopy) [65].

Intracardiac echocardiography (ICE), performed in the catheter laboratory using single-use percutaneous 2D probes introduced transvenously (e.g., via the femoral vein), is also widely used to guide interventions [66]. Its biggest advantage over TOE is the ability to obtain similar echo views without the need for general anaesthesia, with its attendant costs and potential risks. Therefore, ICE has been used in preference to TOE for a number of procedures, including atrial fibrillation [67] and ventricular tachycardia [68] ablation procedures, closure of septal defects [69], TAVI procedures [70] and to guide CRT procedures [71]. However, the significant limitation of ICE is expensive, given that the probes must be disposed of after a single use.

The echocardiologist performing such studies needs to have a sound grasp of normal cardiac and valvular anatomy and combine quick thinking with speedy manual dexterity. This skill set is not necessarily acquired during conventional training. He/she may be called upon to provide urgent information in case of sudden unexplained patient deterioration (e.g., sudden hypotension during TAVI due to entanglement of the delivery system in the mitral subvalvular apparatus causing acute severe mitral regurgitation). The need for such individuals is likely to be restricted to tertiary units, but in large centres one can imagine such a person spending a significant amount of time in the cardiac catheter laboratories. Appropriate training will be required to ensure that we produce cardiologists capable of performing such studies—hence one can envisage the birth of a super-speciality of “interventional echocardiology.”

## 5. Limitations 

Echocardiography does have certain limitations, which merit discussion. The greatest advantage of cross-sectional imaging (i.e., CMR and CCT) over echocardiography is the information available on extracardiac structures. The aorta, aortic arch, pulmonary artery, and pulmonary veins are far more clearly seen by these modalities and are often poorly seen or not seen at all by echocardiography. Thus, assessment of pulmonary veins in patients awaiting an atrial fibrillation ablation procedure, assessment of the aortic arch and descending thoracic aorta in patients with chronic aortic dissection or coarctation, and serial assessment of patients with previous coarctation repair, for example, will generally be performed by CMR or CCT. 

Echocardiography can also suffer from poor image quality, despite the use of ultrasound contrast agents. This is most often seen in patients who are extremely underweight or overweight, but also in patients with chronic airways disease or chest wall deformities (e.g., pectus excavatum). Patients on intensive care units, who are frequently supine and may be mechanically ventilated, also often have challenging TTE images, although TOE can resolve such difficulties. 

## 6. Conclusion

Echocardiography permits accurate assessment of myocardial structure, function and perfusion. Echocardiography is thus in itself a multimodality imaging technique. The field of echocardiography is diverse and encompasses numerous individual techniques—some with established clinical value, whilst others show much promise in research. The expanding role of echocardiography within current clinical practice is underpinned by continual technological advances and justified by the accumulating evidence of clinical value of these techniques when utilised in daily practice.

## Figures and Tables

**Table 1:** A summary table comparing the four main modalities in cardiac imaging.

	Echocardiography	Nuclear cardiology	CMR	CCT
Availability	+++	++	++	++
Cost	+	++	++	++
Ionising radiation	−	++	−	++
Extracardiac information	+	+	+++	+++
Coronary artery assessment	+	−	++	+++
Assessment of systolic function	+++	++	+++	++
Assessment of diastolic function	+++	+	+	+
Assessment of valvular function	+++	−	++	+
Physiological stress testing	+	+	−	−
Pharmacological stress testing	+	+	+	−
Contraindication	−	−	Most implanted devices	−
Limited by renal failure	−	−	+	+
Risk of claustrophobia	−	+	++	+

**Table 2:** Contemporary indications for stress echocardiography.

Indication	Parameter(s) measured
Coronary applications	
Myocardial ischaemia	Wall thickening at rest versus stress
Myocardial viability	Wall thickening at rest, low-dose, and peak stress

Noncoronary applications	
(A) Valve disease	
Asymptomatic severe AS	Exercise-induced change in mean transaortic gradient Exercise-induced change in peak transtricuspid gradient
Low-flow low-gradient AS	LVOT and AoV VTI at rest and low-dose dobutamine stress
Symptomatic mild/moderate MS	Exercise-induced change in mean transmitral and transtricuspid gradients
Symptomatic moderate MR	Exercise-induced changes in EROA and pulmonary pressures
(B) Cardiomyopathy	
HCM	Exercise-induced dynamic LVOT obstruction
DCM	Contractile reserve in response to low-dose dobutamine
ICM	Assessment of global LV contractile reserve Assessment of viability in posterolateral walls (for guiding LV lead placement) Assess exercise capacity objectively Assess inducibility of tachyarrhythmias

(AS: aortic stenosis; MS: mitral stenosis; MR: mitral regurgitation; LVOT: left ventricular outflow tract; AoV: aortic valve; EROA: effective regurgitant orifice area; VTI: velocity time integral; HCM: hypertrophic cardiomyopathy; DCM: dilated cardiomyopathy; ICM: ischaemic cardiomyopathy).
